# Mammalian Resilience Revealed by a Comparison of Human Diseases and Mouse Models Associated With DNA Helicase Deficiencies

**DOI:** 10.3389/fmolb.2022.934042

**Published:** 2022-08-11

**Authors:** Masaoki Kohzaki

**Affiliations:** Department of Radiobiology and Hygiene Management, Institute of Industrial Ecological Sciences, University of Occupational and Environmental Health Japan, Kitakyushu, Japan

**Keywords:** DNA helicase, human disease, mouse model, lifespan - longevity, resilience

## Abstract

Maintaining genomic integrity is critical for sustaining individual animals and passing on the genome to subsequent generations. Several enzymes, such as DNA helicases and DNA polymerases, are involved in maintaining genomic integrity by unwinding and synthesizing the genome, respectively. Indeed, several human diseases that arise caused by deficiencies in these enzymes have long been known. In this review, the author presents the DNA helicases associated with human diseases discovered to date using recent analyses, including exome sequences. Since several mouse models that reflect these human diseases have been developed and reported, this study also summarizes the current knowledge regarding the outcomes of DNA helicase deficiencies in humans and mice and discusses possible mechanisms by which DNA helicases maintain genomic integrity in mammals. It also highlights specific diseases that demonstrate mammalian resilience, in which, despite the presence of genomic instability, patients and mouse models have lifespans comparable to those of the general population if they do not develop cancers; finally, this study discusses future directions for therapeutic applications in humans that can be explored using these mouse models.

## Role of DNA Helicases in Maintaining Genome Integrity

DNA helicase was first discovered in 1970 (Abdel-Monem et al., 1976; Mackay and Linn, 1976) as an enzyme that can move along a nucleic acid as a motor protein that unwinds and unpacks the condensed genomic DNA compacted by chromatin in an ATP-dependent manner (Lusser and Kadonaga, 2003; Clapier et al., 2017). In addition, the ATPases associated with various cellular activities’ (AAA+) domain can drive the conformational changes at the interface of neighboring subunits mediated by ATP binding and hydrolysis, sometimes forming complexes, for efficient remodeling or translocation of target substrates (Singleton et al., 2007). These unwinding and translocation activities of DNA helicase are important for many aspects of genomic metabolism, including DNA recombination, replication, repair, and transcription (Kogoma, 1997), and are indispensable for maintaining genomic integrity, which is tightly related to cancer progression and aging mechanisms (Mohaghegh and Hickson, 2001; Hoeijmakers, 2009). The human genome encodes over 31 DNA helicases and 64 RNA helicases (Umate et al., 2011). DEAD (Asp-Glu-Ala-Asp) box and related DExD/H RNA helicases and DNA–RNA helicases are involved in transcription, resolving transcription–replication conflicts, and G-quadruplex structures (Tanner and Linder, 2001). There are excellent reviews on RNA helicases (Bourgeois et al., 2016), which are not addressed in this review. Human DNA helicases are classified into six superfamilies (SF1–SF6) based on their shared sequence motifs (Gorbalenya et al., 1989; Singleton et al., 2007).

## Human Diseases Associated With Hereditary Mutations in DNA Helicases and Mouse Models

**FIGURE 1:**
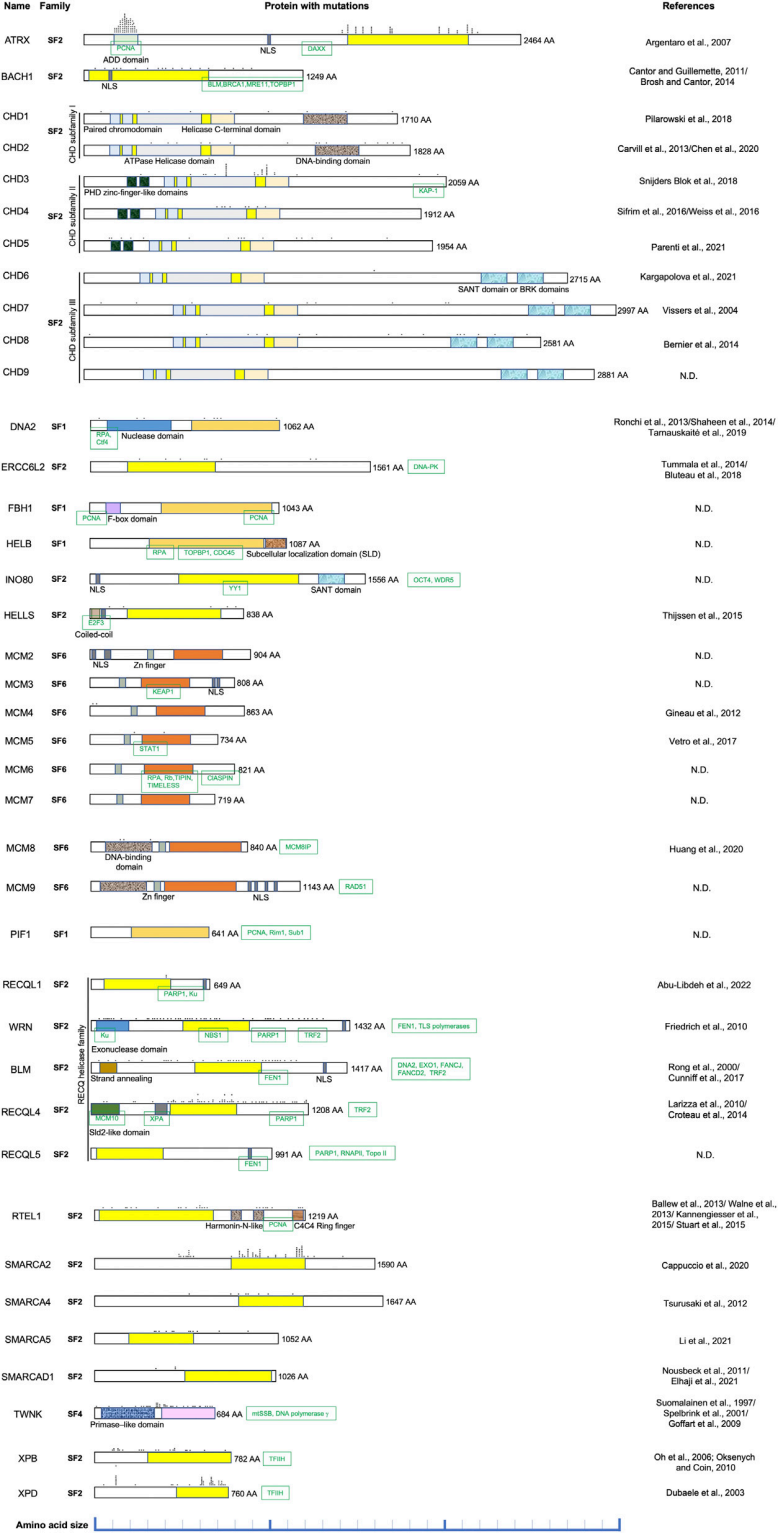
Comparison of DNA helicases with alterations that cause human diseases. DNA helicases are divided into six superfamilies, and they are differently colored as follows: SF1, light orange; SF2, yellow; SF4, light pink; and SF6, orange. The domains characteristics of some proteins are color-coded and the names are written below the domains. The representative interacting proteins are marked with green squares at the interaction site of the DNA helicase of interest. All of the alterations including point mutations, frameshift mutations, missense mutation, nonsense mutations, silent mutations, splice mutations, deletions, insertions, duplications, and repeat elongations are marked with asterisks above the protein structure for simplicity. It is noted that the scale and the position of alterations are not exactly drawn. Please see the references for the exact location and type of each alteration.

**FIGURE 2:**
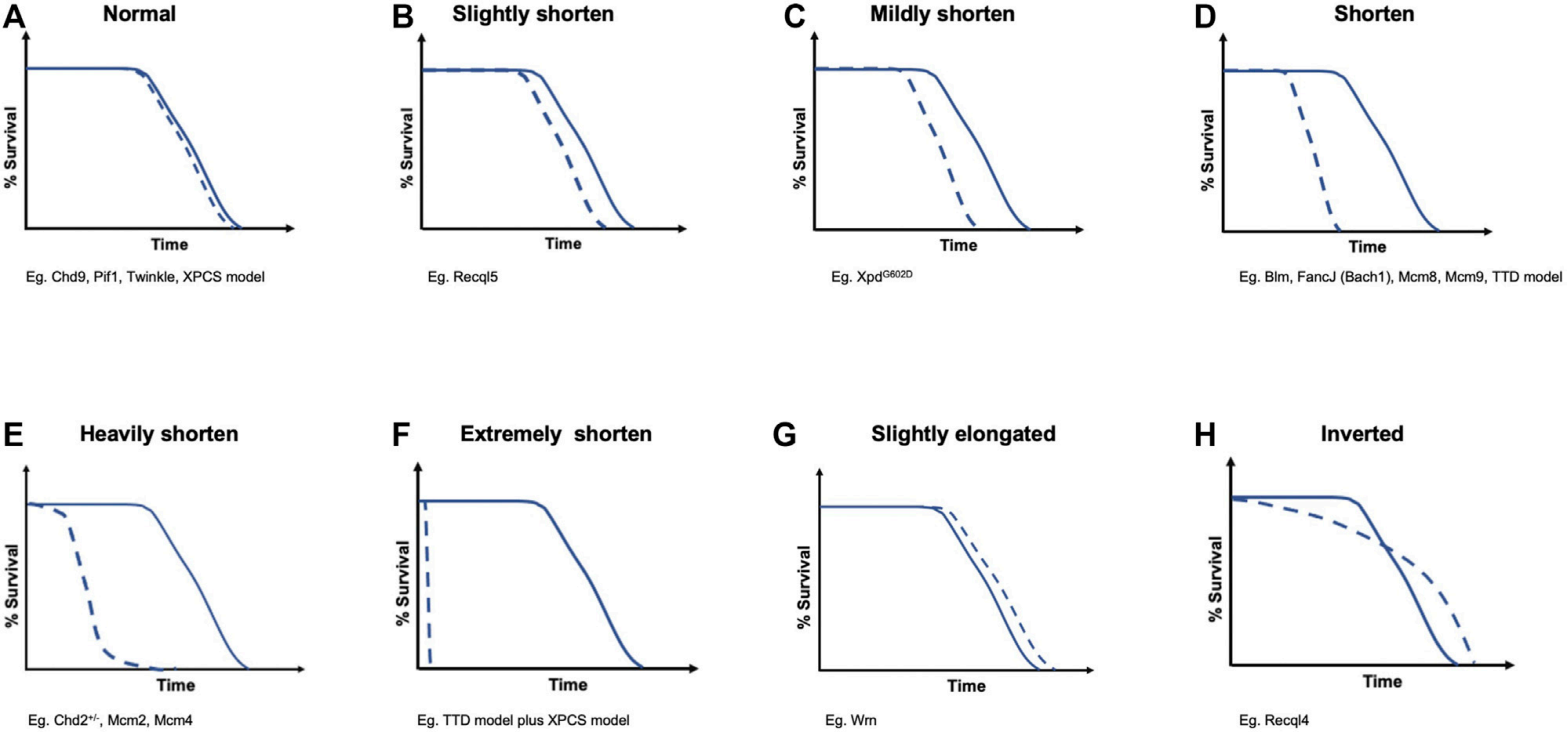
Different survival curves induced by DNA helicase deficiencies. They are categorized into eight groups as follows: **(A)** normal; **(B)** slightly shorten; **(C)** mildly shorten; **(D)** shorten; **(E)** heavily shorten; **(F)** extremely shorten; **(G)** slightly elongated; and **(H)** inverted. These categories correspond to the survival curve items in Table 1

**FIGURE 3:**
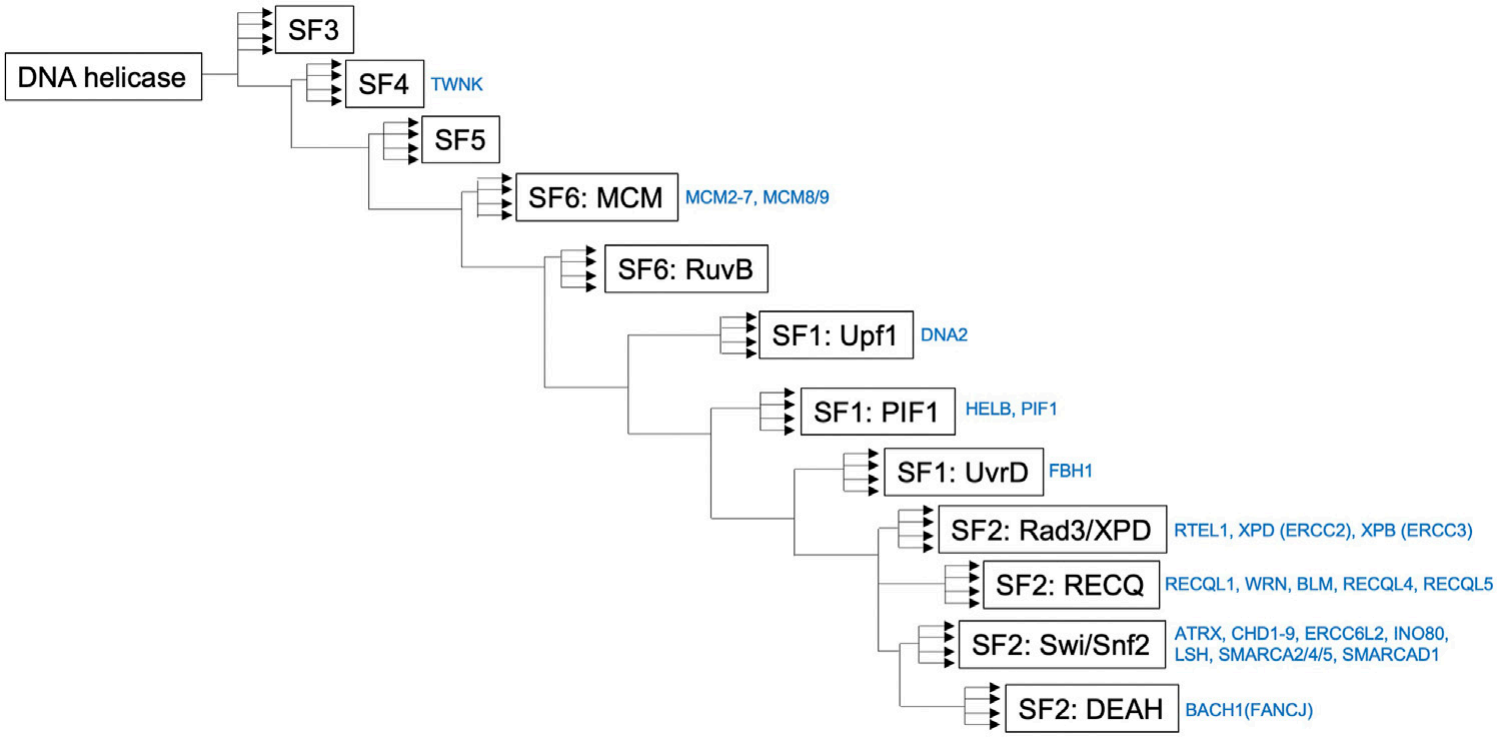
Simplified phylogenetic tree showing the evolutionary relationships of human helicases comprising the major families SF1–6. The figure was made by referring the reported figure by Jackson et al. (2014). It should be noted that a simplified phylogenetic tree does not describe the precise evolutionary time scale, and it can be used as a relative relationship for the DNA helicases presented in this review.

It has been reported that hereditary mutations in DNA helicases can cause human diseases. The RECQ helicase family is one of the most well-known diseases associated with DNA helicase deficiencies (Croteau et al., 2014). The DNA helicase BRCA1-associated C-terminal helicase/BRCA1-interacting protein 1 (BACH1/BRIP1) is inactivated in patients with Fanconi anemia (FA); BACH1 is also known as FA complementation group (FANCJ) (Bridge et al., 2005; Levitus et al., 2005; Levran et al., 2005; Litman et al., 2005). FA is characterized by bone marrow failure, cellular hypersensitivity to DNA interstrand cross-linkers (ICLs), multiple congenital abnormalities, and cancer predisposition (Auerbach, 2009). Xeroderma pigmentosum type B (XPB) and type D (XPD) DNA helicases are involved in transcription-coupled DNA repair (Drapkin et al., 1994; Coin et al., 2007), and mutations in XPB and XPD helicases are found in patients with Cockayne syndrome (CS), trichothiodystrophy (TTD), and xeroderma pigmentosum (XP) (Lehmann A. R, 2001; Kraemer et al., 2007). Due to the biological importance of these diseases, various animal models, mainly using mice, have been established to elucidate their pathogenic mechanisms and show various symptoms related to vital function maintenance, carcinogenesis, and aging. In the following sections, I discuss the diseases caused by deficiencies in each DNA helicase and the mouse models corresponding to these diseases. Some DNA helicases, including Ku70/XRCC6 and PCNA-associated recombination inhibitor (PARI), were omitted due to uncertainty regarding their DNA helicase activity. Representative genome alterations in DNA helicases associated with human diseases are described in Figure 1, which shows specific protein domains and interacting proteins. The relationship between human and mouse is described in Table 1 and shown in the survival curve images in Figure 2. Moreover, a simplified phylogenetic tree of the evolutionary relationships of human helicases comprising the major families SF1–6 is shown in Figure 3 to demonstrate the relative relationships among the DNA helicases presented in this review. The table and figures provide additional information about the mechanisms of diseases caused by DNA helicase deficiencies.

**TABLE 1:** Human diseases, human symptoms, and mouse phenotypes in DNA helicase deficiencies.

Gene	Human disease	Human symptom	Mouse phenotype	Mouse lethality	Postnatal phenotype	Fertility	Survival curve
ATRX	ATR-X syndrome	Alpha-thalassemia/characteristic facial features/severe psychomotor retardation	Hypocellularization of the hippocampus and neocortex/reduced forebrain size	Yes	–	–	–
BACH1	Fanconi anemia	Anemia/high risk for ovarian cancer	Increased MMC sensitivity and lymphoma predisposition	Partially Yes	N.A.	Partially fertile	D (Shortened)
CHD1	Pilarowski–Bjornsson Syndrome	Neurodevelopmental disability	Euchromatin opening in stem cell/epiblast development/HR	Yes	–	–	–
CHD2	Epileptic encephalopathy syndrome	Epileptic encephalopathies/behavioral problems/developmental delay/intellectual disability	**Chd2** ^**+/-**^ scoliosis/shorter lifespan/higher lymphomagenesis	Yes (perinatal)	–	–	E (Heavily shortened)
CHD3	Snijders Blok-Campeau syndrome	Macrocephaly/impaired speech and language	Indispensable for early vascular development	Partially Yes	Died before weaning	–	–
CHD4	Sifrim–Hitz–Weiss syndrome	Intellectual disability/dysmorphisms/heart defects	Impaired blastocyst implantation	Yes	–	–	–
CHD5	Neurodevelopmental syndrome	Behavioral disturbances/epilepsy/intellectual disability	Deregulated spermatogenesis/autism-like characteristics	No	Normal	Infertility in male	N.A.
CHD6	Hallermann-Streiff syndrome-like	Craniofacial dysmorphisms/distinctive facial features/hair and skin abnormalities/short stature/premature aging	Ataxic phenotypes	No	Normal	Fertile	N.A.
CHD7	CHARGE syndrome	Atresia of the choanae/cranial nerve palsies/ear malformations/genital anomalies/intellectual disability/olfactory defects/retarded growth	**Chd7** ^**+/-**^: craniofacial tissues/defects in the ear and heart	Yes	–	–	–
CHD8	Autism spectrum disorder	Impaired brain development and neuron differentiation	**Chd8** ^**+/-**^: ASD-like phenotype/sexually dimorphic behavior	Yes	–	–	–
CHD9	N.D.	N.D.	Loosened chromatin structure in oocytes	No	Normal	Fertile	A (Normal)
DNA2	Seckel syndrome/Mitochondrial myopathy	Primordial dwarfism	**Dna2** ^**+/-**^: telomere DNA damage/chromosome segregation errors/aneuploidy-associated cancers	Yes	–	–	–
ERCC6L2	Bone marrow failure syndrome	Bone marrow failure/craniofacial and cerebellar abnormalities	Decreased in class switch recombination (CSR)	No	N.A.	N.A.	N.A.
FBH1	N.D.	N.D.	Mild induction of Rad51 foci after CPT treatment	No	N.A.	N.A.	N.A.
HELB	N.D.	N.D.	Normal CSR. Important for DNA end resection inhibition	No	Normal	Fertile	N.A.
INO80	N.D.	N.D.	Conditional mIno80 deletion in adults: weight loss and premature death	Yes	–	–	–
LSH	Immunodeficiency, centromeric instability and facial anomalies syndrome	Fatal respiratory/gastrointestinal infections/hypertelorism/epicanthus	Small size/loss of methylation/growth retardation/premature aging	Yes (perinatal)	Die until 1 month	Infertile	–
MCM2-MCM7	Meier-Gorlin syndrome	Growth retardation/adrenal insufficiency, patellar aplasia/hypoplasia, microtia/short stature	Reduced Mcm2, 3, 4 expressions show severe deficiencies in stem cells and higher cancer incidence	Yes	–	–	E (Heavily shortened)
MCM8/9	Primary ovarian insufficiency (POI) syndrome	Reduced reproductive lifespan/myelodysplastic syndrome (MDS) phenotype	Female: primary ovarian insufficiency/development of tumors	No	N.A.	Infertile (MCM9-/- male: fertile)	D (Shortened)
PIF1	N.D.	N.D.	No visible phenotypes/no telomere alteration/mitochondrial myopathy	No	Normal	Fertile	A (Normal)
RECQL1	RECON syndrome	Hypoplastic nose/progeroid facial features/short stature/skin photosensitivity/xeroderma	Elevated spontaneous SCEs/IR sensitivity/not tumor prone	No	Normal	Fertile	N.A.
WRN	Werner syndrome	Premature senescence phenotypes	No apparent senescence phenotypes/increased cardiac fibrosis/perinatal survival disadvantage	No	Perinatally decreased	Fertile	G (Slightly elongated)
BLM	Bloom syndrome	Small size/rash after sun exposure/infertility/immunodeficiency/increased SCEs/cancer predisposition at early ages	**Blm** ^**+/-**^: cancer predisposition/severe anemia	YES/NO	N.A.	Fertile	D (Shortened)
RECQL4	Rothmund-Thomson Syndrome	Congenital bone defect/poikiloderma/osteosarcoma predisposition	**Recql4** ^**HD/HD**^: genome instability/skin abnormality/increased DNA damage in the tail skin and bone marrow	Yes/No(helΔ)	Postnatally decreased	Fertile	H (Inverted)
RECQL5	N.D.	N.D.	Lymphoma and solid tumors predisposition/elevated spontaneous SCEs	No	Normal	Fertile	B (Slightly shortened)
RTEL1	Hoyeraal-Hreidarsson syndrome/idiopathic pulmonary fibrosis	Bone marrow failure/cerebellar hypoplasia/dyskeratosis congenita/enteropathy/immunodeficiency	Normal development/**Rtel1** ^**1A/1A**^: slower replication fork speed and fragile telomeres	Yes	–	–	–
SMARCA2	Nicolaides-Baraitser syndrome	Distal-limb anomalies/distinctive facial morphology/intellectual disability/sparse hair	Weights were significantly heavier than wild-type mice	No	Normal	Fertile	N.A.
SMARCA4	Coffin-Siris syndrome	Coarse facial features/hypoplastic nail/growth deficiency/intellectual disability/microcephaly	**Smarca4** ^**+/**-^: predisposed to exencephaly and tumors	Yes	–	–	–
SMARCA5	Neurodevelopmental syndrome	Postnatal short stature/microcephaly	Required for blastocyst-derived stem cells in embryonic development	Yes	–	–	–
SMARCAD1	Basan syndrome/Adermatoglyphia	Isoform is involved in dermatoglyphic development	Growth retardation/skeletal dysplasia	No	Postnatally decreased	Partially fertile	N.A.
Twinkle	Progressive external ophthalmoplegia (PEO)	Progressive atrophy in brain/sensory axonal neuropathy/neuromuscular symptoms	Respiratory dysfunction/no premature aging/deficiency of cytochrome c oxidase	No	Normal	Fertile	A (Normal)
XPB	TTD, XP, CS, XPCS	Neurodegenerative disorder/premature aging/hypersensitivity to UV light/high skin cancer risk	**Xpb** ^**-/-**^: lethal due to essential transcription initiation function of TFIIH complex	Yes	–	–	–
			**Xpb** ^**Δ43/Δ43**^ **(XPCS model):** UV sensitivity/no aging and tumorigenesis phenotypes	No	Normal	Fertile	A (Normal)
XPD	TTD, XP, CS, XPCS	Neurodegenerative disorder/premature aging/hypersensitivity to UV light/high skin cancer risk	**Xpd** ^**-/-**^: lethal due to the essential transcription initiation function of TFIIH complex	Yes	–	–	–
			**Xpd** ^**R722W/R722W**^ **(TTD model):** Premature aging/osteoporosis/osteosclerosis/cachexia/early graying	No	Growth retardation	Fertile in male/Reduced in female	D (Shortened)
			**Xpd** ^**G602D**^ **(XPCS model)**: Segmental progeria/cachexia/loss of geminal epithelium/increased skin cancer	No	Developmental delay	Fertile	B (Mildly shortened)
XPB+XPD	TTD, XP, CS, XPCS	Neurodegenerative disorder/premature aging/hypersensitivity to UV light/high skin cancer risk	**Xpb** ^**Δ43/Δ43**^ **Xpd** ^**R722W/R722W**^ **(TTD+XPCS model):** Normal development in utero, but failed to grow and died	No	All die within 1–2 days	–	E (Extremely shortened)

N.D, not determined; N.A, not analyzed. See also Figure 2 for the pattern of survival curves.

### ATRX

ATRX belongs to the Snf2 subfamily of Swi/Snf in the superfamily 2 and shows translocation activity on dsDNA as the ATRX–DAXX complex (Xue et al., 2003; Hargreaves and Crabtree, 2011; Byrd and Raney, 2012). Both the helicase activity of ATRX and interaction with DAXX are indispensable for protection from G4-induced stress (Teng et al., 2021). ATRX shares sequences with DNMT3A, DNMT3B, and DNMT3L, which are involved in DNA methylation, and are referred to as the ATRX-DNMT3-DNMT3L (ADD) domain (Argentaro et al., 2007). Both the ADD domain and helicase domains are hotspots for mutations associated with ATR-X syndrome (Figure 1). The ADD domain interacts with PCNA (Juhász et al., 2018), and the DAXX-interacting domain (DID) interacts with death domain-associated protein (DAXX) (Tang et al., 2004), a chaperon protein. These interactions are required for DNA repair synthesis during HR and chromatin remodeling, respectively. Alpha thalassemia X-linked intellectual disability (ATR-X) syndrome shows severe psychomotor retardation, genital abnormalities, alpha-thalassemia, and characteristic facial features (Gibbons et al., 2000). The phenotypes of ATR-X syndromes might result from changes in global transcriptional regulation (Gibbons et al., 1995). ATR-X syndrome changes the DNA hypomethylation pattern at rDNA, and ATRX is required for meiotic organization in mouse oocytes (Gibbons et al., 2000; De La Fuente et al., 2004).

Atrx knockout is embryonic lethal in mice, and conditional Atrx knockout in the forebrain causes extensive hypocellularization of the hippocampus and neocortex with a marked reduction in forebrain size (Bérubé et al., 2005).

### BACH1 (BRIP1 and FANCJ)

BACH1 belongs to the DEAH subfamily of superfamily 2 and shows translocation activity on ssDNA and unwind dsDNA in a 5′-to-3′ direction (Byrd and Raney, 2012). While the M299I mutant protein, a breast cancer variant, can more robustly unwind and translocate DNA than wild-type BACH1 (Sommers et al., 2009), the A349P mutant protein, which is required for DNA repair, loses ATP hydrolysis coupling for the efficient unwinding of forked structures and translocation activity on DNA (Wu et al., 2010). These results suggest that an appropriate helicase activity of BACH1 is necessary for physiological function and tumor suppression. BACH1 exists as either monomer or dimer (Wu et al., 2012). FANCJ is a protein encoded by the BRCA1-interacting protein (BRIP1) gene (Bridge et al., 2005; Levitus et al., 2005) and plays a role in DNA repair by interacting directly with BRCA1 (Cantor et al., 2001). In addition, the C-terminus of BACH1 interacts with BLM, MRE11, and TOPBP1 (Brosh and Cantor, 2014). Indeed, there are many mutations in the sites that interact with these proteins and the helicase domain (Figure 1). Monoallelic carriers of BRIP1 truncation mutations show susceptibility to breast cancer (Seal et al., 2006) and ovarian cancer (Rafnar et al., 2011), similar to those with BRCA gene mutations.

All the reported FA gene knockout mice are viable (Parmar et al., 2009) and have reduced fertility due to impaired gametogenesis (Parmar et al., 2009). Interestingly, single FA gene knockout mice do not show anemia, skeletal defects, or tumor progression (Parmar et al., 2009); however, these knockout mice exhibit accelerated epithelial tumor formation when mated with p53^+/-^ mice (Houghtaling et al., 2005). In line with the characteristics of FA, Brip1 knockout mice are viable and fertile with very small testes, reflecting impaired spermatogonial proliferation (Sun et al., 2016). In contrast, Matsuzaki et al. independently established Fancj knockout mice that exhibit phenotypes distinct from the canonical FA phenotype, in which mitomycin C (MMC) sensitivity and lymphoma predisposition are increased (Matsuzaki et al., 2015).

### CHD1–CHD9

CHD1–CHD9 belong to the Snf2 subfamily of Swi/Snf in superfamily 2 and show translocation activity on dsDNA (Hargreaves and Crabtree, 2011; Byrd and Raney, 2012). The ATPase domain of the Swi/Snf chromatin remodelers resembles the DEAD/H helicases and is required for DNA translocation activity despite no evidence of helicase activity (Hargreaves and Crabtree, 2011). SNF2 superfamily chromodomain helicase DNA-binding (Chd) family enzymes can change chromatin structure during cell metabolism for processes including DNA repair, replication, and transcription and are therefore implicated in human diseases (Marfella and Imbalzano, 2007). Proteins belonging to this family contain double chromodomains, the SNF2 N-terminal (ATPase helicase domain), and helicase C-terminal domains, which are critical for chromatin remodeling activity (Wilson et al., 2021). These CHD family proteins are divided into three subfamilies based on their additional domains, such as the DNA binding, PHD zinc finger-like, BRK (Brahma and Kismet domain), and SANT (Swi3, Ada2, N-Cor, and TFIIIB) domains (Marfella and Imbalzano, 2007) (Figure 1).

#### CHD1

CHD1 (chromodomain helicase DNA-binding protein 1) exists predominantly as a monomer and plays a role in ATP-dependent chromatin assembly (Lusser et al., 2005). Chd1 interacts with Rtf1, a Paf1/RNA polymerase II complex component, while Spt4-Spt5, and Spt16-Pob3 and regulate transcription elongation (Simic et al., 2003). CHD1 also regulates mouse embryonic stem cell pluripotency by opening euchromatin in mice (Gaspar-Maia et al., 2009) through histone H3 methylation (Pray-Grant et al., 2005). CHD1 plays a role in DNA repair by homologous recombination (HR) (Kari et al., 2016). In addition, CHD1 missense mutations cause neurodevelopmental phenotypes, including autism, developmental delay, facial dysmorphic features, and speech apraxia, collectively referred to as Pilarowski–Bjornsson Syndrome (Pilarowski et al., 2018).

Chd1^−/−^ mice show embryonic lethality even in the absence of p53 mutations (Guzman-Ayala et al., 2015).

#### CHD2

The interaction between CHD2 and PARP1 is required for chromatin expansion and H3.3 deposition at DNA damage sites for efficient NHEJ repair (Luijsterburg et al., 2016). CHD2 is implicated in epileptic encephalopathies (Carvill et al., 2013), which are associated with self-induced seizures (Thomas et al., 2015). In addition, CHD2 haploinsufficiency can induce not only epilepsy but also behavioral problems, developmental delay, and intellectual disability (Chénier et al., 2014). CHD2 is required for neural circuit development and long-term memory (Kim et al., 2018).

Homozygous mutations in the Chd2 gene result in perinatal lethality in mice (Marfella et al., 2006). Heterozygous mutations in the Chd2 gene result in decreased neonatal viability and a shorter life span; various organs, including the kidney, are affected in Chd2^+/−^ mice (Marfella et al., 2006). One group reported that Chd2^+/−^ mice develop scoliosis (Kulkarni et al., 2008), and another revealed that these mice exhibit a shorter lifespan, susceptibility to lymphomas and increased extramedullary hematopoiesis (Nagarajan et al., 2009).

#### CHD3

Both CHD3 and CHD4 are factors of the ATP-dependent chromatin remodeling Mi-2/NuRD (nucleosome remodeling deacetylase) complex, which includes HDAC1/2, MTA1/2/3, RbAp46/48, and MDB2/3 (Xue et al., 1998). The SUMO1 interacting motif (SIM) located at the C terminal of CHD3 binds to SUMOylated KRAB-associated protein-1 (KAP-1), which mediates transcriptional regulation and DNA damage repair at specific chromatin regions. This interaction is perturbed when KAP-1 is phosphorylated at serine 824 by ATM after DNA damage for efficient DNA repair (Goodarzi et al., 2011). CHD3 mutations cause Snijders Blok–Campeau syndrome, characterized by macrocephaly and impaired language and speech (Snijders Blok et al., 2018).

Chd3 deletion causes partial embryonic lethality in mice, and all surviving Chd3-knockout mice die before weaning, Chd3 and Chd4 are indispensable for early vascular development (Xie et al., 2020).

#### CHD4

After oxidative damage, CHD4 interacts with DNMT1, DNMT3A, DNMT3B, PARP, PCNA, EZH2, G9a, and OGG1 (Xia et al., 2017). These interactions after oxidative damage are required for CpG island methylation and can maintain epigenetic suppression of tumor suppressor genes. Therefore, a high level of CHD4 is associated with a poor prognosis. De novo mutations in CHD4 induce intellectual disability with distinctive dysmorphisms and congenital heart defects (Sifrim et al., 2016; Weiss, et al., 2016), collectively referred to as Sifrim–Hitz–Weiss syndrome (SIHIWES).

Chd4-deficiency is embryonic lethal in mice due to impaired blastocyst implantation (O'Shaughnessy-Kirwan et al., 2015).

#### CHD5

CHD5 is a component protein of the nucleosome remodeling deacetylase (Mi-2/NuRD) complex (Potts et al., 2011), which is involved in ATP-dependent chromatin remodeling and histone deacetylase activities. CHD5 is expressed in the nervous system and testes (Thompson et al., 2003; Zhuang et al., 2014). Although CHD5 is a tumor suppressor gene frequently altered in many human cancers (Bagchi et al., 2007), patients with CHD5 mutations do not develop tumors (Parenti et al., 2021), suggesting that germline CHD5 alterations do not increase cancer risk. CHD5-deficient patients demonstrate dominant neurodevelopment disorders, including behavioral disturbances, epilepsy, and intellectual disability (Parenti et al., 2021).

Chd5 knockout mice exhibit and autism-like phenotypes (Pisansky et al., 2017) and male infertility due to decreased spermiogenesis (Zhuang et al., 2014).

#### CHD6

CHD6 is expressed in various mouse tissues and colocalizes with RNA polymerase II during mRNA synthesis (Lutz et al., 2006). CHD6 interacts with the PPAR complex and Nrf2, a transcription factor regulating oxidative stress response (Surapureddi et al., 2002; Nioi et al., 2005). A *de novo* CHD6 missense mutation was recently identified in a patient clinically presenting with Hallermann–Streiff syndrome, a premature aging disorder characterized by craniofacial and dental dysmorphisms, distinctive facial features, hair and skin abnormalities, and short stature (Kargapolova et al., 2021).

Chd6^Exon12-/Exon12-^ mice are deficient in conserved ATPase domain expanding exons 12–19 and are viable but sterile with no obvious pathological phenotypes apart from ataxia (Lathrop et al., 2010).

#### CHD7

CHD7 interacts with transcription factors (Basson and van Ravenswaaij-Arts, 2015) and the BMP signaling pathway nuclear mediators SMAD1/SMAD5/SMAD8 (Liu et al., 2014). The CHD7 helicase gene has been implicated in CHARGE syndrome (Vissers et al., 2004), which is characterized by multiple symptoms, including atresia of the choanae, cranial nerve palsies, ear malformations, genital anomalies, intellectual disability, olfactory defects, and retarded growth (Sanlaville and Verloes, 2007).

Heterogeneous Chd7-mutated mice exhibit many features of CHARGE syndrome, such as craniofacial tissues and defects of the ear and heart (Bosman et al., 2005). Chd7 knockout is embryonic lethal in mice due to severe defects in the development of many tissues, including BMP signaling-regulated cardiogenic processes (Hurd et al., 2007; Liu et al., 2014).

#### CHD8

CHD8 is a chromatin remodeling regulator of many genes, including p53 and beta-catenin (Nishiyama et al., 2004; Nishiyama et al., 2009). CHD8 can regulate neuronal development associated with autism spectrum disorder (ASD), which is associated with chromatin modification and transcriptional regulation (Bernier et al., 2014; Sugathan et al., 2014). CHD8, KATNAL2, and SCN2A have been revealed by exome sequencing to be autism risk factors in humans (Iossifov et al., 2012; Neale et al., 2012; O'Roak et al., 2012a, 2012b; Sanders et al., 2012).

Homozygous Chd8 knockout is embryonic lethal in mice (Nishiyama et al., 2004; Nishiyama et al., 2009), but Chd8^+/−^ mice are viable and exhibit phenotypes resembling ASD (Katayama et al., 2016). Recently, Chd8^+/N2373K^ mice reflecting human ASD were found to show sexually dimorphic behavior changes associated with neuronal activity and gene expression (Jung et al., 2018).

#### CHD9/CReMM

Chd9 is highly expressed in oocytes and is associated with promoters in osteogenic cells (Shur et al., 2006).

Chd9 is involved in the chromatin loosening of growing oocytes, which is required for the acquisition of totipotency after fertilization in mice (Ooga et al., 2018). However, another group reported that Chd9 knockout mice are fertile and show no apparent phenotypes (Alendar et al., 2020).

### DNA2

DNA2 possesses endonuclease activity. DNA2 belongs to the DEAD-like subfamily in superfamily 1 and can unwind dsDNA in a 5′-to-3′ direction. DNA2 forms oligomers and acts as a helicase in replication (Budd et al., 1995). DNA2 helicase/nuclease interacts with AND-1 (Ctf4) FANCD2, FEN1, PIF1, and RPA (Bae et al., 2003; Karanja et al., 2012; Sparks et al., 2020) and is indispensable for Okazaki fragment processing during DNA replication (Budd and Campbell, 1997; Bae et al., 2001). In addition, DNA2 is involved in mitochondrial DNA maintenance (Budd et al., 2006) and the double-strand break (DSB) repair pathway (Zhu et al., 2008). Human and yeast DNA2 can bind to telomeric G4 structures within ssDNA for G4 structure cleavage and unwind intermolecular G4 structures (Masuda-Sasa et al., 2008). A homogenous truncation mutation in DNA2 likely causes a form of primordial dwarfism, namely, Seckel syndrome (Shaheen et al., 2014; Tarnauskaitė et al., 2019). DNA2 mutations also induce mitochondrial myopathies (Ronchi et al., 2013).

Homozygous Dna2 knockout is embryonic lethal in mice, and heterozygous Dna2-deficient mice have increased telomere DNA damage and chromosome segregation errors that cause aneuploidy. As a result, Dna2-deficient mice develop aneuploidy-associated cancers and have dysfunctional telomeres (Lin et al., 2013).

### ERCC6L2

ERCC excision repair 6 like 2 (ERCC6L2) belongs to the Snf2 subfamily of Swi/Snf in the superfamily 2 (Byrd and Raney, 2012). DNA damage induction can induce the translocation of ERCC6L2 from the cytoplasm to the mitochondria and nucleus (Tummala et al., 2014). ERCC6L2 interacts with MRI/CYREN, a cell cycle regulator of NHEJ, and regulates orientation-specific joining of broken ends during class switch recombination (CSR), which relies on the helicase activity of ERCC6L2 (Liu et al., 2020). Homozygous truncating mutations of the ERCC6L2 gene have been identified by exome sequencing in patients with bone marrow failure (BMF) and neurological dysfunction (Tummala et al., 2014; Bluteau et al., 2018). BMF is characterized by hematopoietic abnormalities and a predisposition to cancer, as typified by patients with FA (Kutler et al., 2003), and BMF patient cells with ERCC6L2 mutations show an impaired DNA repair pathway resulting from DNA-dependent protein kinase (DNA-PK) impairment and increased genomic instability similar to that observed in FANCG patient cells in response to ICLs (Tummala et al., 2018).

Ercc6l2^−/−^ mice were viable and showed decreased nonhomologous end-joining (NHEJ)-associated CSR (Liu et al., 2020).

### FBH1

The F-box DNA helicase 1 (FBH1) belongs to DNA helicase superfamily 1, and unwinds dsDNA in a 3′-to-5′ direction. The FBH1 gene is conserved from fission yeast to vertebrates (Kim et al., 2002). FBH1 interacts with PCNA *via* two distinct motifs (Bacquin et al., 2013). The F box domain at the N-terminus of FBH1 suggests that it is an E3 ubiquitin ligase. Although fbh1 is indispensable for viability in the absence of rqh1 (RECQ helicase in humans) or srs2 (an antirecombinase that can remove rad51 recombinase from ssDNA) DNA helicases (Krejci et al., 2003; Veaute et al., 2003), single fbh1 knockout results in mild phenotypes in yeast (Morishita et al., 2005; Osman et al., 2005). FBH1-knockout chicken B lymphocyte DT40 cells show no prominent phenotypes except mildly increased sister chromatid exchanges (SCEs), and an additive increase in SCE induction and a synergistic sensitivity to camptothecin (CPT) are observed in the absence of BLM (Kohzaki et al., 2007).

Consistently, no apparent phenotypes except mild induction of Rad51 foci after CPT treatment and micronuclei induction after topoisomerase II inhibitor treatment are observed in Fbh1 knockout mice (Laulier et al., 2010).

### HELB

DNA helicase B (HELB) belongs to helicase superfamily 1B and shows translocation activity on ssDNA in a 5′-to-3′ direction (Hormeno et al., 2022). The 5′-to-3′ translocation activity of HELB is required for preventing DNA end resection in HR (Tkáč, J et al., 2016). The helicase activity of HELB is quite weak without assisting force (Hormeno et al., 2022). When HELB binds to RPA, HELB translocation activity is increased but unwinding is inhibited (Hormeno et al., 2022). HELB helicase plays a role in the early stages of replication initiation (Matsumoto et al., 1995). Indeed, HELB interacts not only with RPA but also with TOPBP1 and CDC45 (Guler et al., 2012; Gerhardt et al., 2015).

Although, mHelb knockout mice are fertile and do not show clear phenotypes under unchallenged conditions, MEF cells from Helb knockout mice show decreased replication rates after hydroxyurea treatment, suggesting that mHelb is involved in the recovery from replication stress (Tkáč, J et al., 2016).

### INO80 (INOC1)

The chromatin remodeling complex inositol requiring mutant 80 (INO80) belongs to the Ino80 subfamily of Swi/Snf in superfamily 2 and shows translocation activity on dsDNA (Hargreaves and Crabtree, 2011; Byrd and Raney, 2012). INO80 is a core component of the chromatin remodeling INO80 complex, and this large INO80 complex forms a functional dimer, similar to Snf2h (Willhoft et al., 2017). In fact, INO80 has translocation activity along DNA at the H2A–H2B dimer interface for efficient H2A.Z exchange by displacing DNA (Brahma et al., 2017). In addition, the INO80 complex shows DNA helicase activity and binds Holliday junctions and replication forks *in vitro* (Shen et al., 2000). Therefore, INO80 is involved in DNA repair, replication, and transcriptional regulation (van Attikum and Gasser, 2009).

mIno80 knockout mice die during early embryogenesis, and conditional depletion of mIno80 in adult mice results in premature death and weight loss (Min et al., 2013).

### LSH (Also Known as HELLS, PASG, or SMARCA6)

Lymphoid-specific helicase (LSH) belongs to the Snf2 subfamily of Swi/Snf in superfamily 2 and shows translocation activity on dsDNA (Hargreaves and Crabtree, 2011). The ATPase of LSH for remodeling activity can be stimulated by nucleosomes through the formation of the LSH-CDC7A complex (Jenness et al., 2018). Like CHD1-9, the ATPase domain of LSH is required for DNA translocation activity despite no evidence of helicase activity (Hargreaves and Crabtree, 2011). LSH interacts with DNMT3b, E2F3, and CDC7A and plays roles in DNA methylation (Zhu et al., 2006), transcription (von Eyss et al., 2012), and NHEJ repair (Jenness et al., 2018; Unoki et al., 2019), respectively. LSH is involved in chromatin packaging, DNA methylation, lymphoid development, and stem cell proliferation (Dennis et al., 2001). Mutations in lymphoid-specific helicase (HELLS) and cell division cycle associated 7 (CDCA7) can cause a genetically heterogeneous autosomal recessive disorder referred to as centromeric instability and facial anomalies (ICF) syndrome, which is characterized by life-threatening immunodeficiency (Thijssen et al., 2015). However, tumorigenesis induced by LSH deficiency has rarely been reported (Lee et al., 2000; Yano et al., 2004). In contrast, overexpression of LSH can promote invasive carcinoma (Yang et al., 2019).

In stark contrast to human patients, Hells-knockout mice die perinatally; thus, Hells is considered an essential gene for mouse viability (Geiman et al., 2001). Lsh-knockout mice exhibit perinatal lethality with substantial loss of genome-wide methylation (Dennis et al., 2001). Mice with Pasg mutations show growth retardation and premature aging (Sun et al., 2004).

### MCM2–MCM7

Minichromosome maintenance protein complex (MCM) helicases 2–7 contain the AAA + domain and belong to superfamily 6 (Singleton et al., 2007). MCM 2–7 form a hexamer complex that plays a role in DNA replication initiation (Perkins and Diffley, 1998). Only MCM2 and MCM3 have NLS (nuclear localization signals) among the six MCM helicases (Kimura et al., 1996). The protein interaction of MCM2-7 with specific proteins may determine the specialized functions of these MCM2-7 helicases. For example, MCM3 interacts with Keap1, resulting in an antioxidant response (Tamberg et al., 2018). The interaction between STAT1 and MCM5 is required for IFNγ-induced transcriptional activation (Zhang et al., 1998). MCM6 interacts with RPA, Rb, Timeless/Tipin, and Claspin for efficient replication initiation (Komata et al., 2009; Numata et al., 2010).

Patients with partial MCM4 deficiency show growth retardation, adrenal insufficiency, and selective NK deficiency (Gineau et al., 2012). Recently, it has been reported that MCM5 is involved in Meier–Gorlin syndrome (MGS), characterized by patellar aplasia or hypoplasia, microtia, and short stature (Vetro et al., 2017). Fewer than 100 patients with MGS have been reported since the first description of the condition by Meier in 1959 and Gorlin in 1975 (de Munnik et al., 2015). Interestingly, recessive mutations in prereplication complex (pre-RC) genes, including ORC1, ORC4, ORC6, CDT1, CDC6, and CDC45, have been reported to be involved in MGS (Bicknell et al., 2011), suggesting that factors involved in DNA replication initiation play a role in suppressing MGS symptoms.

Nearly all the reported knockouts of pre-RC genes in mice are embryonic lethal (Yoshida et al., 2001; Kwon et al., 2011; Skarnes et al., 2011; Okano-Uchida et al., 2018), suggesting that replication initiation is biologically indispensable. Specifically, mice with reduced Mcm2, Mcm3, and Mcm4 expression levels show severe deficiencies in stem and progenitor cells due to replication stress and a higher cancer incidence (Pruitt et al., 2007; Shima et al., 2007; Chuang et al., 2010; Alvarez e al., 2015). In contrast, overexpression of pre-RC genes may be prognostic in several cancers (Cai et al., 2018; Li et al., 2018), suggesting that proper expression of pre-RC genes is considered necessary for homeostasis.

### MCM8 and MCM9

MCM8/9 have an AAA + domain and belong to superfamily 6, similar to MCM2-7. However, MCM8 and MCM9 form an oligomeric complex that, unlike MCM2-7, is not essential for DNA replication initiation (Maiorano et al., 2005; McKinzey et al., 2021). Recently, it was reported that nonconventional NLS motifs exist at the C-terminus of MCM9 (McKinzey et al., 2021). The MCM8-MCM9 complex is required for HR induced by ICLs in chicken B lymphocyte DT40 cells (Nishimura et al., 2012). MCM8 and MCM9 interact with MCM8IP and RAD51, respectively, for efficient HR repair (Park et al., 2013; Huang et al., 2020).

Mutations in MCM8-9 can cause primary ovarian insufficiency (POI), a syndrome characterized by reduced reproductive lifespan (Desai et al., 2017). Therefore, the MCM8-MCM9 complex plays a role in meiosis mediated by HR. Recently, MCM9 mutations were found to be associated with germline predisposition to early onset inherited colorectal cancers (Goldberg et al., 2021).

MCM8-MCM9-deficient mice exhibit gametogenesis deficiency, and MCM8 knockout female mice develop ovarian tumors, although MCM8/9-knockout mice are viable (Hartford et al., 2011; Lutzmann et al., 2012). Biallelic mutations in MCM8 and MCM9 in female mice result in POI (Lutzmann et al., 2012). MCM8/9-deficient mice accumulate hematopoietic cell DNA damage with age and demonstrate an increased frequency of myeloid tumors in a p53-dependent manner (Lutzmann et al., 2019).

### PIF1

PIF1 belongs to helicase superfamily 1B and is an ATP-dependent 5′–3′ DNA helicase. Like other SF1 helicases, ScPif1 exists as a monomer in solution (Lahaye et al., 1993), and ScPif1 is dimerized upon ssDNA binding (Barranco-Medina and Galletto, 2010). Interestingly, dimerized ToPif1 inhibits its helicase activity (Dai et al., 2021), suggesting a diversified helicase regulation mechanism among DNA helicases. ScPif1 interacts with PCNA (Wilson et al., 2013), Rim1, a mitochondrial ssDNA-binding protein (Ramanagoudr-Bhojappa et al., 2013), and Sub1, a G4-binding factor (Lopez et al., 2017) for recombination-coupled DNA synthesis, function in mtDNA metabolism (Ramanagoudr-Bhojappa et al., 2013) and suppressing genome instability at transcriptionally formed G4 DNA, respectively. Pif1 can resolve G-quadruplex structures (Sanders, 2010) and prevent telomere elongation (Schulz and Zakian, 1994).

In contrast, mPif1-knockout mice are viable and show no visible phenotypes and no telomere length alteration (Snow et al., 2006), suggesting that telomere protection is compensated by other DNA helicases, such as Dna2, in mice. Bannwarth et al. revealed that Pif1 knockout mice develop mitochondrial myopathy (Bannwarth et al., 2016).

### RECQ Helicases

RECQ helicases belong to the RECQ-like subfamily of superfamily 2, show translocation activity on ssDNA (Kitano, 2014), and unwind dsDNA in a 3′–5′ direction (Byrd and Raney, 2012). In vertebrates, five SF2 superfamily RECQ helicases have similar helicase domains (Croteau et al., 2014). However, these proteins express different RecQ C-terminal (RQC) (Bernstein et al., 2003), helicase and RNase D–like C-terminal (HRDC) (Liu et al., 1999), exonuclease (Shen et al., 1998), sld2-like (Sangrithi et al., 2005; Matsuno et al., 2006) and Zn knuckle (Marino et al., 2013) domains, and nuclear localization sequences (NLS) (Kitao et al., 1998). Interestingly, only RECQL4 has a NLS at the N terminus of the helicase domain (Burks et al., 2007). In addition, RECQ helicases share interacting proteins, such as RAD51 and RPA, but some interactions are missing in some RECQ helicases. For example, all RECQ helicases except BLM interact with PARP1 (Croteau et al., 2014). In addition, five RECQ helicases have different binding partners involved in their function (Lu and Davis, 2021). Therefore, the outcomes and symptoms associated with deficiencies in these five RECQ helicases are very different. To date, no clear reports on patients with mutations in RECQL5 (RECQ5) have been published.

#### RECQ1 (RECQL1)

RECQL1 is the smallest gene in the RECQ family (Puranam and Blackshear, 1994) and forms either monomers or dimers (Vindigni and Hickson, 2009). RECQL1 helicase is related to the risk for some diseases, including breast cancer, and patient treatment outcomes (Cybulski et al., 2015; Sun et al., 2015; Viziteu et al., 2017). Recently, two patients with progeroid facial features, a hypoplastic nose, xeroderma and skin photosensitivity with short stature were identified and had the same missense mutation (Ala459er) located in the zinc-binding domain of the RECLQ1 gene, referred to as RECON (RECqlONe) (Abu-Libdeh et al., 2022).

Recql1 knockout mice show a generally normal phenotype, but MEFs obtained from Recql1 mice show genomic instability, such as chromosomal breakage, aneuploidy, SCEs (Sharma et al., 2007).

#### WRN (RECQL2)

Werner syndrome (WS), a rare autosomal-recessive disease, is characterized by premature senescence phenotypes in young adults (Goto et al., 1992; Friedrich et al., 2010). Among RECQ family helicases, only WRN has an exonuclease domain at the N-terminus (Huang et al., 1998). WRN forms hexamers and trimers (Perry et al., 2006). Recently, synthetic lethality between WRN and mismatch repair (MMR) has been identified by several groups (Chan et al., 2019; Kategaya et al., 2019; Lieb et al., 2019; van Wietmarschen et al., 2020), suggesting that WRN can regulate DNA repair pathway mechanisms promoting cancer cell survival.

Notably, Wrn-knockout mice show no apparent senescence-like phenotype and a normal lifespan (Lebel and Leder, 1998; Lombard et al., 2000; Ichikawa et al., 2002). In contrast, Terc^−/−^Wrn^−/−^ mice exhibit premature aging phenotypes, including alopecia, cataracts, diabetes, hair graying, and osteoporosis (Chang et al., 2004; Du et al., 2004), suggesting that the abundant telomere reserve in mice results in phenotypic differences compared with that in other mammals. Wrn-deficient mice exhibit accelerated mortality in the absence of the p53 gene (Lombard et al., 2000). Wrn^hel/hel^ mice die from increased cardiac fibrosis rather than cancers or infections (Massip et al., 2006).

#### BLM (RECQL3)

Like WRN, BLM forms hexamers and trimers (Karow et al., 1999). Bloom syndrome (BLM) patients are characterized by a proportional small size and a face rash that develops during Sun exposure (Rong et al., 2000; Cunniff et al., 2017). BLM syndrome patients show cancer predisposition at early ages (occurring in the second decade of life, on average), impaired fertility, and immune deficiency (Ellis et al., 1995). A significant increase in SCEs in BLM patient cells has been well recognized (Chaganti et al., 1974) and can induce mutagenesis (Carrano et al., 1978; Wilson and Thompson, 2007).

Several laboratories have established Blm-knockout mice, and some Blm-knockout mice show embryonic lethality (Chester et al., 1998; Ichikawa et al., 2002), chromosomal instability, and severe anemia (Chester et al., 1998). Viable Blm-knockout mouse strains show cancer predisposition (Luo et al., 2000), even when the knockout is heterogeneous (Goss et al., 2002).

#### RECQ4 (RECQL4)

Rothmund Thomson syndrome (RTS) is characterized by growth retardation with skeletal defects and congenital poikiloderma (Larizza et al., 2010). Defects in the RECLQ4 gene cause type II RTS, characterized by congenital bone defects, poikiloderma, and osteosarcoma predisposition (Kitao et al., 1999; Wang et al., 2003). RECQL4 exists as a dimer (Suzuki et al., 2009). RECQL4 is the least well-characterized among RECQ helicases in terms of function because it has an N-terminal budding yeast Sld2 domain, which is essential for DNA replication initiation (Sangrithi et al., 2005; Matsuno et al., 2006; Tanaka et al., 2007; Zegerman and Diffley, 2007), and complete RECQL4 knockout is lethal in vertebrates (Abe et al., 2011). RECQL4 knock-in human pre-B leukemia Nalm-6 cells are highly sensitive to radiation and cisplatin and show replication elongation defects after ionizing radiation (Kohzaki et al., 2012). Although RECQL4 is involved in several DNA repair pathways, such as HR (Lu et al., 2016), NHEJ (Shamanna et al., 2014), and single-strand annealing (SSA) (Kohzaki et al., 2020), a functional assay using siRNA targeting the RECQL4 helicase domain significantly reduces the expression of the Sld2 domain (Kohzaki et al., 2020), which is essential for survival and requires careful phenotyping.

Ichikawa et al. first confirmed RECQL4 lethality (Ichikawa et al., 2002). Recql4 knockout mice established by another group demonstrated embryonic lethality (Hoki et al., 2003), confirming the essential role of the Sld2 domain at the N-terminus of the Recql4 gene. Viable Recql4-deficient (Recql4^HD/HD^) mice exhibit genomic instability, skin abnormalities and increased intestinal adenomas when adenomatous polyposis coli (Apc) is heterogeneously absent (Mann et al., 2005). Recql4 ^HD/HD^ mice exhibit senescence with increased DNA damage in tail fibroblasts, sparser tail hair, and significantly fewer blood cells in the bone marrow (Lu et al., 2014). In contrast, we did not observe such a significant reduction in blood cells in Recql4 ^HD/HD^ mice, and the function of Recql4 in hematopoiesis should be carefully tested in the future. Interestingly, viable Recql4 ^HD/HD^ mice show a pleiotropic phenotype in which more than half show a dramatically reduced risk of age-related mortality (approximately seven-fold) compared to that of wild-type mice throughout their lifespan (Kohzaki et al., 2021).

#### RECQL5 (RECQ5)

RECQL5 can interact with RNAPII (Aygün et al., 2008; Izumikawa et al., 2008) and harbors two RNAPII interaction domains for different phenotypes (Islam et al., 2010). RECQL5 forms a monomer (Garcia et al., 2004). RECQL5 inhibits transcription and affects transcription elongation to suppress genomic rearrangements and transcription stress (Aygün et al., 2009; Saponaro et al., 2014). Despite these important biological features, no human patients with RECQL5 mutations have been identified to date.

Recql5-knockout mice show a predisposition to various types of cancer, including lymphoma and solid tumors (Hu et al., 2007), and exhibit elevated levels of SCEs (Hu et al., 2005), suggesting that RECQL5 is a tumor suppressor gene.

### RTEL1

Regulator of telomere elongation helicase 1 (RTEL1), a human novel helicase-like (NHL) gene, belongs to the Rad3/XPD subfamily of helicase superfamily 2 (Byrd and Raney, 2012), and oligomerized RTEL1 unwinds dsDNA in a 5′ to 3′ direction (Ghisays et al., 2021). RTEL1 helicase interacts with PCNA through the PCNA interacting protein (PIP) motif and regulates telomere length and HR to maintain genomic integrity (Bai et al., 2000; Ding et al., 2004; Vannier et al., 2013). Furthermore, the telomeric G4 unwinding activity of RTEL1 depends on the RTEL1-PCNA interaction, suggesting that RTEL1 can resolve the telomeric G4 structure of replication forks during DNA replication (Vannier et al., 2013). Biallelic RTEL1 mutations have been observed in patients with congenital dyskeratosis (Ballew et al., 2013; Walne et al., 2013). Heterozygous RTEL1 mutations are associated with idiopathic pulmonary fibrosis with telomere shortening (Kannengiesser et al., 2015; Stuart et al., 2015; Newton et al., 2016). Several RTEL1 mutations have also been identified in patients with Hoyeraal–Hreidarsson syndrome symptoms, including cerebellar hypoplasia, enteropathy, hypocellular bone marrow failure, B-/NK-cell lymphopenia, and immunodeficiency (Speckmann et al., 2017).

Homozygous Rtel1 knockout is embryonic lethal in mice (Ding et al., 2004). Mice with Rtel1 mutations (Rtel1^IA/IA^) in the IA PIP box, which is required for PCNA interaction, are viable and show normal development and morphology (Vannier et al., 2013). However, Rtel1 ^IA/IA^ mice demonstrate accelerated tumorigenesis in the absence of p53 (Vannier et al., 2013), suggesting that Rtel1 is a potent tumor suppressor gene, which has been previously revealed by a genome-wide association study that identified it as a glioma susceptibility locus (Shete et al., 2009).

### SMARCA2 (BRM)

Multi-subunit chromatin remodeling complex Switch/Sucrose nonfermentable (SWI/SNF) consists of helicase/ATPase catalytic subunits, SWI/SNF-related matrix-associated actin-dependent regulator of chromatin subfamilies A2 (SMARCA2) and A4 (SMARCA4) (Wilson and Roberts, 2011). Therefore, SMARCA2 and SMARCA4 belong to the Swi/Snf subfamily in superfamily 2 and show translocation activity on dsDNA (Hargreaves and Crabtree, 2011; Byrd and Raney, 2012). Like other SWI/SNF family members, the ATPase domain of SMARCA2/4/5/SMARCAD1 are required for DNA translocation activity despite no evidence of helicase activity (Hargreaves and Crabtree, 2011). Heterozygous missense mutations in SWI/SNF-related matrix associated, actin-dependent regulator of chromatin, subfamily A, member 2 (SMARCA2) cause Nicolaides–Baraitser syndrome, characterized by distal-limb anomalies, distinctive facial morphology, intellectual disability, and sparse hair (Van Houdt et al., 1997). Although it has recently been reported that mutations in SMARCA2 outside of the helicase domain can also cause a new recognizable syndrome with intellectual disability and blepharophimosis distinct from Nicolaides–Baraitser syndrome (Cappuccio et al., 2020), it remains unclear whether these mutations affect ATP-dependent helicase activity.

Mice lacking Smarca2 are viable, fertile and significantly heavier than wild-type mice (Reyes et al., 1998). Smarca2 plays a greater role than Smarca4 in cell proliferation, although Smarca2 and Smarca4 are partially redundant (Reyes et al., 1998).

### SMARCA4 (BRG1)

Along with SMARCB1, ARID1A, and ARID1B mutations, SMARCA4 mutations are associated with Coffin–Siris syndrome, a rare congenital anomaly syndrome characterized by coarse facial features, hypoplastic nail of the fifth finger and/or toe, growth deficiency, intellectual disability, and microcephaly with an autosomal dominant inheritance mechanism (Tsurusaki et al., 2012). Recently, mutations in SMARCA4 have been identified in a group of undifferentiated thoracic malignancies, which are distinct from lung carcinomas and transcriptionally related to BAF-deficient sarcomas (Le Loarer et al., 2015).

In stark contrast to Smarca2, Smarca4 knockout is early embryonic lethal in mice, and Smarca4 heterozygotes are predisposed to exencephaly and tumors (Bultman et al., 2000).

### SMARCA5 (SNF2H)

SMARCA5 (SNF2h) is an ATPase-dependent helicase subunit of the Imitation SWItch (ISWI) complex and regulates DNA accessibility by sliding the histone octamer (Fan et al., 2003). Thus, SMARCA5 belongs to the Snf2 subfamily of Swi/Snf in superfamily 2 and shows translocation activity on dsDNA (Hargreaves and Crabtree, 2011; Byrd and Raney, 2012). Only a minor fraction of SMARCA5 can bind to chromatin and translocate the post-translational modified nucleosome, while the majority of SMARCA5 is highly mobile (Erdel et al., 2010). De novo or rare heterozygous variants of SMARCA5 in 12 patients were found to cause a neurodevelopmental syndrome with recurrent dysmorphic features, such as postnatal short stature associated with mild developmental delay and microcephaly (Li et al., 2021).

Homozygous Smarca5 knockout is early embryonic lethal in mice (Stopka and Skoultchi, 2003).

### SMARCAD1 (ETL1)

SMARCAD1 (ETL1) is a member of the SNF2 family, belongs to the Snf2 subfamily of Swi/Snf in superfamily 2 and shows translocation activity on dsDNA (Korn et al., 1992). SMARCAD1 is as chromatin remodeler recruited to DSBs for efficient resection for subsequent HR repair (Costelloe et al., 2012). A skin-specific SMARCAD isoform regulates dermatoglyphic development (Nousbeck et al., 2011). Recently, two patients with SMARCAD1 variants showed symptoms of Basan Syndrome characterized by absent or reduced sweating, congenital adermatoglyphia, neonatal acral bullae, and transient congenital facial milia (Elhaji et al., 2021).

Smarcad1 knockout mice are viable but exhibit decreased viability, impaired fertility, and skeletal dysplasia (Schoor et a., 1999).

### Twinkle (TWNK) Mitochondrial DNA Helicase

TWNK belongs to helicase superfamily 4 and unwinds DNA in a 5′-to-3′ direction. Replication of mitochondrial DNA (mtDNA) depends on the replisome containing DNA helicase TWNK, mitochondrial ssDNA-binding protein (mtSSB), and DNA polymerase G (POLG). TWNK forms stable hexamers for helicase loading (Farge et al., 2008). TWNK is indispensable for mitochondrial helicase activity required for the synthesis of D-loop strands to complete mtDNA replication (Milenkovic et al., 2013). Some patients with TWINKLE mutations develop adult-onset progressive external ophthalmoplegia (PEO), progressive atrophy of the cerebellum, brain stem, spinal cord, sensory axonal neuropathy, and neuromuscular symptoms (Suomalainen et al., 1997; Spelbrink et al., 2001; Goffart et al., 2009).

Mice with Twinkle mutations demonstrate key features observed in the muscles of PEO patients, including progressive respiratory dysfunction and cytochrome C oxidase deficiency in distinct neuronal populations, at 1 year of age. Interestingly, these mice do not show premature aging, suggesting that progressive respiratory chain dysfunction and mtDNA deletions are not strong aging accelerators (Tyynismaa et al., 2005).

### XPD (ERCC2)/XPB (ERCC3)

XPD belongs to the Rad3/XPD subfamily in superfamily 2, shows translocation activity on ssDNA and unwinds dsDNA in a 5′-to-3′ direction (Fan et al., 2008; Byrd and Raney, 2012). In contrast, XPB also belongs to superfamily 2 but can unwind DNA in a 3′–5′ direction. These opposite unwinding directions are indispensable for nucleotide excision repair (NER). Both XPB and XPD are components of the TFIIH transcription complex in NER repair. CS caused by mutations in CSA or CSB is a rare autosomal recessive neurodegenerative disorder characterized by an impaired nervous system, growth failure, lipodystrophy, photosensitivity without skin cancer predisposition, and a very short (<20 years) life expectancy due to premature aging (Calmels et al., 2018). Both CSA and CSB are involved in base excision repair (BER) and transcription-coupled nucleotide excision repair (TC-NER) (van der Horst et al., 1997; Stevnsner et al., 2002).

XP is an autosomal recessive disorder characterized by a deficiency in NER and hypersensitivity to UV light (Lehmann et al., 2011). As a result, XP patients are extremely sensitive to sunlight and have a dramatically increased skin cancer risk, and their life expectancy is approximately 30 years less than that of the general population.

TTD is an autosomal recessive disorder characterized by brittle hair, a Sun-sensitive form of severe neurodevelopment, developmental abnormalities, photosensitivity, skin abnormality, maternal pregnancy complications, and reduced life span with high mortality rates at a young age (Faghri et al., 2008). Mutations in the XPB (ERCC3) and XPD (ERCC2) genes can lead to TTD, XP, CS, and XP combined with CS (XPCS) (Coin et al., 1999; Dubaele et al., 2003; Oh et al., 2006; Oksenych and Coin, 2010).

There are several mouse models of XPD and XPB. XPD is essential for cell viability. Deletion of XPD in mice is embryonic lethal (de Boer et al., 1998b). Mice with Xpd mutated at amino acid 722 (Xpd^R722W/R722W^) reflected TTD patients (de Boer et al., 1998a). TTD model mice have compromised transcription and show many symptoms, including premature aging, osteoporosis, osteosclerosis, cachexia, early graying, sterility, and a shortened lifespan (de Boer et al., 2002). TTD mice show prominent premature aging phenotypes and a shortened lifespan without mutation accumulation in the kidney and liver (Dollé, et al., 2006), suggesting that increased genomic instability induced by DNA repair-deficiency is not a prerequisite for lifespan shortening in mice. Mice with the Xpd mutation (G602D) are considered xeroderma pigmentosum/Cockayne syndrome (XPCS) model mice and show segmental progeria, cachexia, progressive loss of germinal epithelium, and skin cancer-predisposition due to unusual NER dysfunction (Andressoo et al., 2006). The phenotypes of these XPCS-disorder mice are similar to those of TCR-deficient Csa^−/−^ mice, which show white matter microglial activation (Jaarsma et al., 2011), suggesting that NER plays a role in neuronal maintenance.

Xpb knockout is embryonic lethal in mice (Andressoo et al., 2009). Xpb mice with the last 43 amino acids deleted (Xpd^Δ43/Δ43^) were established as a model for XPCS, and Xpd^Δ43/Δ43^ mice are fertile with no overt phenotypes such as growth retardation, premature aging, and cancer predisposition, while Xpd^Δ43/Δ43^ mice are sensitive to UVB irradiation of the skin (Andressoo et al., 2009). Interestingly, when a second TFIIH helicase mutation is present, Xpd knockout mice are viable but die during the neonatal period and exhibit increased DNA damage (Andressoo et al., 2009), suggesting that the basal TFIIH transcription function during development *in utero* is sufficient without these two helicases but insufficient for postnatal development.

## Mechanisms of DNA Helicases that Cause Human Diseases

**FIGURE 4:**
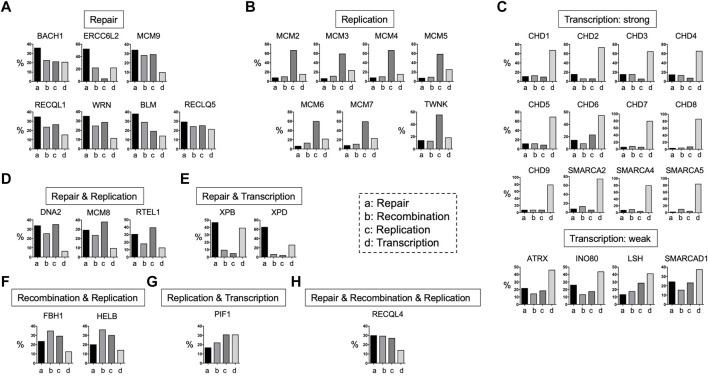
Functions of DNA helicases. DNA helicases have four main functions: DNA repair, DNA recombination, DNA replication, and transcription based on the PubMed search in June of 2022. They are divided into several categories based on the ratios of these four functions. **(A)**: Repair, **(B)**: replication, **(C)**: transcription, **(D)**: repair and replication, repair and transcription, **(E)**: repair and transcription, **(F)**: recombination and replication, **(G)**: replication and transcription, and **(H)**: repair and recombination and replication.

DNA helicases can act on various substrates at different biological steps through different interacting proteins (Figure 1). In addition, DNA helicases have functional preferences in DNA repair, recombination, replication, and transcription (Figure 4). Therefore, the outcomes induced by DNA helicase deficiencies result in variable phenotypes in humans and mice (Table 1). Common mechanisms associated with DNA helicase deficiency involve DNA metabolism, including DNA repair, recombination, replication, repair, and transcription. For example, patients with progeroid syndromes caused by DNA helicase deficiencies, including FA, CS, TTD, and WRN, often also exhibit neurodegenerative phenotypes, suggesting that some DNA helicases are required for neuronal development and/or homeostasis (Maynard et al., 2015). DNA helicases that result in embryonic lethality when deficient must be involved in the critical steps of DNA metabolism (Table 1). Considering the case of Ino80-knockout mice, it is likely that the INO80 helicase is also required for human embryonic development but has never been recognized as a human syndrome. In contrast, some DNA helicase-deficient mice demonstrate a nearly normal phenotype, and some DNA helicases have not been associated with human diseases. Notably, mice deficient in DNA helicases such as Chd9, Fbh1, and Pif1, which are not associated with human syndromes, are viable, fertile, and show normal postnatal growth (Table 1), suggesting that deficiencies in these DNA helicases are easily compensated by other DNA helicases *in vivo*; thus, it is challenging to detect mutations associated with human disease. Alternatively, a human disease may not relate to the overlap of helicase functions and may simply be due to the relatively rare frequency of heterozygous mutations in the general population. Deficiencies in some DNA helicases, such as RECQL5, are not linked to human diseases. It is tempting to speculate that populations with RECQL5 mutations may have mild, if any, phenotypes, and the mutations are less likely to be detected than other hereditary diseases associated with easily detectable symptoms, including BLM and WRN. Thus, the population with RECQL5 mutations may have a lifespan similar to that of the general population. As a result, it is expected that most patients who have germline mutations with unnoticed phenotypes may be missed until they present with cancers or other age-related diseases and undergo precise genetic testing.

DNA mutations as a consequence of DNA metabolism errors resulting from DNA helicases deficiencies are now accurately determined using sequencing technologies (Schumacher et al., 2021; Cagan et al., 2022). These studies revealed that somatic mutations accumulate with age in all mammals. Therefore, either germline mutations or spontaneous somatic mutations occurring with aging resulting from DNA helicase deficiencies may affect complex physiological systems, including epigenetic regulation, gene regulatory networks, and noncoding region regulation, leading to diverse phenotypes (Sen et al., 2016; Anastasiadou et al., 2018; Vijg and Dong, 2020).

## Possible Reasons for the Difference Between Human Diseases and Mouse Models

Experimental mouse models are indispensable tools for understanding human diseases due to their uniform genetic background, short lifespan, and physiologic characteristics similar to those of other mammalian species. However, there are several limitations to using mice as model animals. First, it is debatable to what extent mice with a uniform genetic background can reflect the fairly heterogeneous human population. Second, mice and humans may have different physiological circadian rhythm functions due to the reversal of the active phase between day and night. In addition, mice are susceptible to experimental conditions and the rearing environment, including pathogen-free conditions, temperature, and the microbiome (Ngyen et al., 2015). Third, different mouse strains have different phenotypes, and an appropriate mouse strain should be selected. Forth, neutrophils are the predominant white blood cells in humans, while lymphocytes are the predominant white blood cells in mice in the hematopoietic system (Kohnken et al., 2017). As a result, a high proportion of lymphomagenesis often masks tumor induction in mouse models, such as models of breast cancer (Evers and Jonkers, 2006). Fifth, there are some intrinsic differences in the gut microbiota between mice and humans, and the gut microbiota is associated with some human diseases (Nguyen et al., 2015). Last, differences between the innate and adaptive immune systems and inflammatory responses in humans and mice should be considered (Mestas and Hughes, 2004; Seok et al., 2013). However, Takao and Miyakawa have argued that the inflammatory response data between humans and mice was highly correlated when the data used by Seok et al. was analyzed by sophisticated and commonly used (Seok et al., 2013; Takao and Miyakawa, 2015), suggesting that care should be taken when choosing analytical methods to assess mouse phenotypes.

Due to these limitations, some mutations in DNA helicases, such as ATRX and CDH genes, in humans that result in viable individuals with clinical features are embryonic lethal in mice. On the other hand, the characteristics of FA patients, including cancer predisposition and anemia, are absent in FA gene-knockout mice (Parmar et al., 2009). Generally, p53 gene deficiency combined with DNA helicase knockout results in severe phenotypes and enhanced cancer progression in mice (Lombard et al., 2000; Houghtaling et al., 2005; Mann et al., 2005; Vannier et al., 2013; Lutzmann et al., 2019), apart from in Chd1-knockout mice (Guzman-Ayala et al., 2015). Thus, in the absence of DNA helicases in the mouse, a short-lived laboratory animal, p53 is essential for maintaining genomic integrity and preventing cancers.

Comprehensive whole-genome sequencing using 16 mammalian species revealed that aging-associated somatic mutation accumulation is mainly attributed to the deamination of 5-methylcytosine in humans, while somatic mutation accumulation in mice is highly induced by oxidative DNA damage (Cagan et al., 2022). Indeed, mouse cells are more sensitive to oxidative DNA damage than are human cells (Coppé et al., 2010). Such a difference in mutation accumulation between humans and mice may be one of the reasons for the phenotypic differences. In addition, DNA helicase deficiency may alter germline and/or somatic integrity, affecting complicated gene regulatory networks or noncoding networks (Anastasiadou et al., 2018; Vijg and Dong, 2020); the response of these networks may differ among species.

## Mammalian Resilience in DNA-Metabolizing Enzyme Disorders

When we consider the relationship between aging and lifespan, we can refer to several aging hypotheses, such as the “mutation accumulation” hypothesis (Medawar, 1952), “antagonistic pleiotropy” hypothesis (Williams, 1957), and “disposable soma” hypothesis (Kirkwood, 2005), which explain the accumulation rate of multiple types of damage in complex networks with maintenance and repair functions in a stochastic and plastic manner. These hypotheses suggest that it is important for long-lived mammals to minimize the damage accumulation associated with tumorigenesis induced by DNA metabolism deficiency (Marmot et al., 2013). Recently, an aging model in which deleterious parental alleles were negatively selected to achieve early life mortality revealed that overall mortality throughout the lifespan was affected (Kinzina et al., 2019). This early life mortality can also be considered a bottleneck period in mouse development. In general, the more severe the bottleneck postnatal growth retardation in transgenic mice is, the higher the probability of lifetime risk of death in mouse experiments will be (Figures 2B–F) (Andressoo et al., 2009). Moreover, the developmental origins of health and disease (DOHaD) concept has been proposed, in which environmental influences during early development can affect the risks of physiological processes, including chronic diseases and noncommunicable diseases (NCDs), later in life (Hanson and Gluckman, 2014). Interestingly, Recql4-deficient mice have a higher postnatal mortality rate than wild-type mice, but the risk of death decreases dramatically with age, and the Kaplan–Meier survival curve is reversed later in life (Figure 2H) (Kohzaki et al., 2021). This suggests that resilience against accumulating mutations and genomic instability with aging in mammals may exist under certain physiological conditions.

The direct comparison between germline and somatic mutation rates in humans and mice reveals that somatic mutations have an approximately two orders of magnitude higher frequency than germline mutations (Milholland et al., 2017), suggesting that strong negative selection aggressively occurs to eliminate deleterious mutations in the germline. Moreover, somatic mutations negatively correlate with lifespan in mammals (Cagan et al., 2022). These somatic mutations that accumulate with aging are considered a consequence of DNA metabolism failure, including DNA helicases and DNA polymerases, and the DNA damage theory of aging is a convincing aging theory (Figures 2B–F) (Hoeijmakers, 2009; Freitas and de Magalhães, 2011). Indeed, the accumulation of chromosomal aberrations with aging, such as loss of the Y chromosome (LOY), is associated with a higher risk for cancer, a shorter lifespan, and other age-related diseases (Dumanski et al., 2016; Loftfield et al., 2018; Thompson et al., 2019). Consistently, it was proposed that DNA DSB repair efficacy correlates with maximum lifespan based on results obtained from 18 rodents with varying lifespans (Tian et al., 2019). Therefore, less efficient DNA repair results in genomic instability increases, which can accelerate the aging rate (Figures 2B–F). However, it remains to be determined why Recql4-deficiency is associated with resilience against aging in mice with higher levels of genomic instability and DNA damage than wild-type mice (Figures 2G,H) (Mann et al., 2005; Lu et al., 2014; Kohzaki et al., 2021).

Cancer predisposition syndromes caused by germline mutations in DNA polymerase epsilon (POLE) and delta (POLD) and the DNA glycosylase MUTYH gene, a BER factor, have recently been reported (Robinson et al., 2021a; 2021b). Patients with these mutations show a markedly increased risk for colorectal and endometrial cancer with several-fold higher mutation rates than those of the general population. Interestingly, these patients do not show overt premature aging or age-related diseases, and many patients survive until the late decades, comparable with the unaffected population. These phenotypes are reminiscent those of RTS patients, who can live for as long as the general population if they do not develop cancers (Larrizza et al., 2010). Importantly, these phenotypes in human patients are similar to those in mice with germline mutations of Pole, Pold, and homozygous knockout of Mutyh that show a normal lifespan with no apparent premature aging phenotypes in untreated conditions (Venkatesan et al., 2007; Albertson et al., 2009; Li et al., 2017). This is also the case with other DNA repair-deficient mice in which increased genomic instability is not correlated with a shortened lifespan (Figure 2A) (Dollé et al., 2006). Given that OGG1 and MUTYH are important for BER to remove accumulated endogenous oxidative DNA lesions such as 7,8-dihydro-8-oxoguanine (8-oxoG), the lack of overt phenotypes in untreated Mutyh knockout mice is surprising, particularly considering that mutations accumulate during aging. These findings indicate that the elevated mutation burdens associated with POLD/POLE and MUTYH deficiency can be mitigated by unknown factors, resulting in resistance to small insertion and deletion (ID) mutations and single-base substitution (SBS) mutations throughout life and no apparent aging-associated biological dysfunction. Many factors are involved in DNA repair pathway responses to various types of endogenously or exogenously induced DNA damage. Therefore, the loss of some DNA helicases may be efficiently compensated by other factors and may not cause an obvious phenotype such as Fbh1 and Pif1 knockout mice (Table 1). It is tempting to speculate that the system leading to the mammalian resilience system (MRS) may be activated when a certain level of mutations or certain conditions of genomic instability occur. Increased apoptosis due to DNA damage accumulation can be triggered to exclude these deleterious products in organs (Dollé et al., 2006). However, this possibility appears unlikely because programmed cell death, including apoptosis, declines with age (Tower, 2015), and p53-mediated apoptosis and/or senescence contribute to aging (Herranz and Gil, 2018). Alternatively, lifespan-limiting factors could be counteracted by factors that can elongate the lifespan. Mammalian aging and longevity processes consist of a complex variety of biological functions that are strongly influenced by environmental factors and may not be improved by a single or small number of factors or genes (Schumacher et al., 2021). However, several studies have focused on lifespan extension in mice, investigating caloric restriction and lipid metabolism and models such as dwarf and transgenic mice, including Atg5-overexpressing mice (Liang et al., 2003; Pyo et al., 2013; Johnson and Stolzing, 2019; Singh et a., 2019). These mice may be useful to identify lifespan-limiting factors to develop methods for elongating lifespan regardless of the accumulation of somatic mutations with age, which is accelerated by DNA-metabolizing enzyme disorders.

## Future Perspectives

Deficiencies in some DNA helicases, such as BLM, FANCJ, RECLQ4, WRN, and XPD (Table 1) induce cancers in humans. On the other hand, overexpression of some of these DNA helicases is implicated in various types of carcinogenesis. Therefore, appropriate expression of DNA-metabolizing enzymes, including DNA helicases and DNA polymerases, is indispensable for maintaining homeostasis, particularly in later life, when physiological deficiencies are likely to be more pronounced in long-lived mammals such as humans (Hanson and Gluckman, 2014). Such balanced homeostasis can be easily affected in shot-lived mammals such as mice in the absence of p53, a guardian of genomic stability. Since accumulating somatic mutations with age and mammalian resilience are a homeostatic balance system regulated by complex and multiple physiological systems and influenced by environmental conditions, mouse strains that show unconventional lifespans or environmental settings would be useful to uncover largely unknown MRS. Understanding the mechanisms of mammalian resilience could yield a better understanding of healthy life expectancy for applied use in the future.
